# Extrinsic Sources of Cholinergic Innervation of the Striatal Complex: A Whole-Brain Mapping Analysis

**DOI:** 10.3389/fnana.2016.00001

**Published:** 2016-01-22

**Authors:** Daniel Dautan, Husniye Hacioğlu Bay, J. Paul Bolam, Todor V. Gerdjikov, Juan Mena-Segovia

**Affiliations:** ^1^MRC Anatomical Neuropharmacology Unit, Department of Pharmacology, University of OxfordOxford, UK; ^2^Department of Neuroscience, Psychology and Behaviour, University of LeicesterLeicester, UK; ^3^Department of Anatomy, School of Medicine, Marmara UniversityIstanbul, Turkey; ^4^Center for Molecular and Behavioral Neuroscience, Rutgers UniversityNewark, NJ, USA

**Keywords:** striatum, nucleus accumbens, cholinergic brainstem, cholinergic interneurons, acetylcholine

## Abstract

Acetylcholine in the striatal complex plays an important role in normal behavior and is affected in a number of neurological disorders. Although early studies suggested that acetylcholine in the striatum (STR) is derived almost exclusively from cholinergic interneurons (CIN), recent axonal mapping studies using conditional anterograde tracing have revealed the existence of a prominent direct cholinergic pathway from the pedunculopontine and laterodorsal tegmental nuclei to the dorsal striatum and nucleus accumbens. The identification of the importance of this pathway is essential for creating a complete model of cholinergic modulation in the striatum, and it opens the question as to whether other populations of cholinergic neurons may also contribute to such modulation. Here, using novel viral tracing technologies based on phenotype-specific fluorescent reporter expression in combination with retrograde tracing, we aimed to define other sources of cholinergic innervation of the striatum. Systematic mapping of the projections of all cholinergic structures in the brain (Ch1 to Ch8) by means of conditional tracing of cholinergic axons, revealed that the only extrinsic source of cholinergic innervation arises in the brainstem pedunculopontine and laterodorsal tegmental nuclei. Our results thus place the pedunculopontine and laterodorsal nuclei in a key and exclusive position to provide extrinsic cholinergic modulation of the activity of the striatal systems.

## Introduction

The striatum (STR) plays a key role in learning, memory and motor control (Mink, 1996) and is implicated in a variety of neurological disorders. It constitutes the main input nucleus of the basal ganglia, receiving major afferents from the cortex, the thalamus and the dopaminergic midbrain (Bolam et al., 2000). Predominantly composed of GABAergic projection neurons, the striatum contains a small proportion of cholinergic interneurons (CIN) that, despite their low number, have been proposed to provide the striatum with one of the highest concentrations of cholinergic markers in the brain (Macintosh, 1941; Hebb and Silver, 1961; Woolf et al., 1984; see review by Lim et al., 2014).

Historically, the evidence suggested that CINs are the principal source of cholinergic markers in the striatum (Bennett et al., 2000; Wang et al., 2006; Ding et al., 2010; Goldberg et al., 2012; see review by Calabresi et al., 2000) but our recent analysis of brainstem cholinergic neurons [pedunculopontine nucleus (PPN) and laterodorsal tegmental nucleus (LDT)] using anterograde conditional tracing, identified that these neurons target the striatum (Dautan et al., 2014; see also Saper and Loewy, 1982; Smith and Parent, 1986; Hallanger and Wainer, 1988; Nakano et al., 1990). Similar to CINs, projections from the brainstem largely avoid striosomes, have extensive ramifications and give rise to both symmetric and asymmetric synapses, although their proportions vary (Dautan et al., 2014). Notably, brainstem cholinergic projections exhibit a rostrocaudal topographical organization, where the PPN (rostral brainstem, associated with motor circuits) predominantly targets the dorsolateral striatum, and the LDT (caudal brainstem, associated with limbic circuits) targets the dorsomedial striatum and nucleus accumbens. Thus, the extrinsic cholinergic innervation of the striatum follows a functional motor-to-limbic gradient, but the extent to which this also involves other cholinergic structures is not known, particularly in light of recently developed technologies that allow increased sensitivity to detect phenotype-specific labeling of axons.

Cholinergic innervation of the brain is widely distributed and predominantly originates from eight anatomically segregated nuclei: medial septum (Ch1), the vertical limb of the diagonal band of Broca (Ch2), the horizontal limb of the diagonal band of Broca (Ch3), the nucleus basalis of Meynert (Ch4), the PPN (Ch5), the LDT (Ch6), the medial habenula (Ch7) and the parabigeminal nucleus (Ch8) (Mesulam et al., 1983a,b; Mufson et al., 1986; Mesulam and Geula, 1988; Mesulam, 1990). While there is some evidence of connectivity between neurons contained within these regions and the striatum (e.g., Wall et al., 2013), it is uncertain whether these neurons are cholinergic and thus contribute to the cholinergic innervation of the striatum. Because of the potential implications of additional sources of acetylcholine in the striatum, we investigated this possibility by targeting all eight brain cholinergic nuclei using a combined anterograde and retrograde analysis of cholinergic neurons and created maps of their axonal distributions. Our results support the previous findings of a direct cholinergic brainstem projection to the striatum, and demonstrate that the PPN and LDT constitute the only external source of acetylcholine to the striatum.

## Materials and Methods

### Animals

Adult (250–350 g) male Long Evans (LE) wild types (*n* = 8) and Chat::Cre^+^ (*n* = 19; Witten et al., 2011) rats were used for all experiments. Rats were maintained on a 12 h light/dark cycle (light on 07:00 am) and had *ad libitum* access to water and food. All procedures were performed in accordance with the Society of Neuroscience policy on the use of animals in neuroscience and the Animals (Scientific Procedures) Act, 1986 (UK) under the authority of a Project Licence approved by the Home Office and the local ethical committee of the University of Oxford.

### Stereotaxic Injections

All surgical procedures were performed during deep isoflurane anesthesia (2% in O_2_, IsoFlo, Schering-Plough). ChAT::Cre^+^ rats were injected in each hemisphere with adeno-associated virus serotype 2 carrying the fusion genes for the enhanced yellow fluorescent protein (AAV2-EF1a-DIO-eYFP) or mCherry protein (AAV2-EF1a-DIO-mCherry; Gene Therapy Center Virus Vector Core, University of North Carolina). The viral vectors were injected in a volume of 300 nl for the forebrain cholinergic structures (Ch1 to Ch4) to avoid spreading over contiguous cholinergic structures, whereas a volume of 500 nl was used for the other cholinergic nuclei (Ch5 to Ch8). The injection sites were randomized for hemisphere and fluorescent reporter. Viral injections were delivered in eight different locations corresponding to the eight cholinergic groups described by Mesulam et al. (1983a,b) using the following stereotaxic coordinates (from bregma, in mm; DV ventral to the dura): Ch1 (medial septum) AP +0.7, ML +0.2, DV −4.5; Ch2 (vertical limb of the diagonal band) AP +0.5, ML +0.4, DV −7.5; Ch3 (horizontal limb of the diagonal band) AP +0.1, ML +1.6, DV −8.5; Ch4 (nucleus basalis of Meynert) AP +0.9, ML +2.5, DV −7.0; Ch5 (PPN) AP −7.3, ML +1.8, DV −7.2; Ch6 (LDT) AP −8.5, ML +1.0, DV −6.0; Ch7 (medial habenula) AP −3.5, ML +0.3, DV −4.0; and Ch8 (parabigeminal nucleus) AP −4.5, ML +4.3, DV −5.5 (Paxinos and Watson, 2007).

For the retrograde tracing studies, wild-type LE rats were injected bilaterally with cholera toxin b (Ctb, 2.5% in distilled water, 400 nl over 10 min, Sigma-Aldrich) and Fluorogold (FG, 2.0% in distilled water, 300 nl over 10 min, Fluorochrome) in dorsal striatum and nucleus accumbens. Injections sites, hemispheres and tracers were randomized across animals. Injections were delivered in the dorsal striatum and the nucleus accumbens using the following stereotaxic coordinates (from bregma, in mm; DV ventral to the dura): dorsal striatum AP +0.5, ML +2.1, DV −4.5; nucleus accumbens AP +1.5, ML +1.8, DV −6.7.

For all experiments, injections were made using 1 μl syringes (Neuros 7001, Hamilton) at a rate of 50 nl/min and left to diffuse for 5 min before retraction of the syringe. Approximately 4 weeks following AAV injections, or 10–15 days following tracers injections, rats were humanely euthanized using a lethal dose of pentobarbital (>200 mg/kg, i.p.) and perfused transcardially with 0.05 M PBS, pH 7.4 (approximately 50 ml over 5 min), followed by 300 ml of 4% w/v paraformaldehyde in phosphate buffer (0.1 M, pH 7.4) containing 0.1% glutaraldehyde (TAAB Laboratories) over about 20 min. Brains were stored at 4°C until sectioning.

### Immunohistochemistry

Brain blocks were formed using 2% agarose gel in PBS (Agarose, BIO-41025, Bioline). Coronal or parasagittal sections were cut at 50 μm thickness in PBS using a vibrating microtome (VT1000S, Leica). For each experiment, the sites of injection were verified by fluorescent microscopy and only those with on-target injections were processed further. For the anterograde tracing study, one section every 300 μm of the entire brain was analyzed. Sections were incubated overnight in a solution containing antibodies against green fluorescent protein (GFP, which also detects eYFP; 1:1000 dilution, raised in rabbit, A21311, Invitrogen), mCherry (1:500, raised in mouse, Millipore), choline acetyltransferase (ChAT; 1:500, raised in goat, Millipore) and tyrosine hydroxylase (TH, in order to define striatal borders; 1:500, raised in chicken, Abcam). The antibodies were diluted in 1% normal donkey serum (NDS) and 0.03% Triton X-100 in PBS. After several washes in PBS, the sections were incubated for a minimum of 4 h in secondary antibodies (all raised in donkey, Jackson Immunoresearch) conjugated to one of the following fluorophores: Alexa Fluor 488 (1:1000), CY5 (1:1000), CY3 (1:1000) or AMCA (1:500).

For the retrograde tracer injections, all sections that included each of the cholinergic nuclei (Ch1–Ch8) were collected. Following an overnight incubation with antibodies against ChAT (1:500) and Ctb (1:500, raised in mouse, Abcam), sections were washed in PBS and then incubated in Cy3- or Cy5-conjugated secondary antibodies (1:1000 and 1:500, respectively; raised in donkey, Jackson Immunoresearch). For FG detection, no additional processing was required.

The processed sections were mounted on slides using Vectashield and then examined in a confocal microscope (LSM-510, Zeiss) at two magnifications (10×, 0.32 NA and 20×, 0.8 NA), using the corresponding filters (504 nm for FG and YFP, 560 nm for Cy3 and mCherry, and 650 nm for Cy5). Brightness and contrast of captured images were adjusted in Photoshop (Adobe Systems). AAV-injected sections were contoured and fully scanned on a single Z-stack using StereoInvestigator (MicroBrightField; 10×, 0.25 NA). For each scanned site, the top and bottom of the section were manually delimited based on the section surface and single pictures were captured (2048 × 1056 pixel resolution) at 10 μm below the surface of the section to ensure that the antibody completely penetrated the section (frame size 860 × 660 μm). Scanning sites were selected using the StereoInvestigator system incorporating a XYZ stage controller and a 25% overlap was selected to facilitate reconstruction. To avoid photobleaching, an inter-acquisition interval of 50 ms was applied during scanning. Exposure time was automatically adapted for every site in order to keep the same intensity threshold.

### Analysis and Quantification of the Distribution of Cholinergic Axons

Scans from AAV-injected brains were reconstructed off-line based on the 2D serial section reconstruction module of StereoInvestigator. Scans were then overlapped with outlines of the rat brain atlas (Paxinos and Watson, 2007) with the aid of the TH and ChAT immunostaining. Injection sites with GFP cell body expression less than 30% of the total number of ChAT-positive neurons within the diffusion area, or with positive soma located outside the borders of the nucleus (for contiguous cholinergic structures), were excluded from the analysis.

The density of AAV-positive fibers in each nucleus was qualitatively assessed off-line. Representative levels for each main brain nuclei/region were scored using a predetermined density rating. The relative density was scored 4+ for many labeled fibers covering approximatively more than 50% of the selected image surface (“very dense”). Nuclei were labeled as 3+ or 2+ when projections covered approximatively more than 25% (“dense”) or less than 25% (“moderate”) of the surface, respectively. Structures were noted 1+ (“few”) when only few terminals were visible. All brains were scanned and only nuclei presenting similar scores in at least two animals were considered. Fibers without terminals or arborization, defined as fibers *en-passage*, were excluded from the mapping.

### Analysis of the Distribution of Retrogradely Labeled Neurons

Brains injected with retrograde tracers were sectioned and scanned as described above. Parasagittal and coronal sections that included the striatum and all cholinergic nuclei were examined. Single optical sections containing cholinergic neurons were obtained at high resolution (20×, 0.8 NA, step size 1 μm, 2056 × 1056 pixels) for the fluorescent channels corresponding to the labeling of ChAT^+^, FG^+^ and Ctb^+^ neurons. Analysis of the distribution of retrogradely labeled neurons was carried out using StereoInvestigator functions: the cholinergic nuclei borders were outlined based on ChAT-immunolabeling using *contour* tools, an overlay projection of the Z-stack was obtained based on *average intensity* tools, colocalization of ChAT, Ctb and FG was quantified using the *markers* plugin. A minimum of two single optical sections were examined. Neurons within the borders of each cholinergic nucleus were classified as follows: (1) ChAT^+^/Ctb^+^; (2) ChAT^+^/FG^+^; (3) ChAT^-^/Ctb^+^; and (4) ChAT^-^/FG^+^. Data were confirmed in a minimum of four animals where Ctb and FG injections were alternated between dorsal striatum and nucleus accumbens.

## Results

### Conditional Labeling and Mapping of Cholinergic Axons

**Figure 1:**
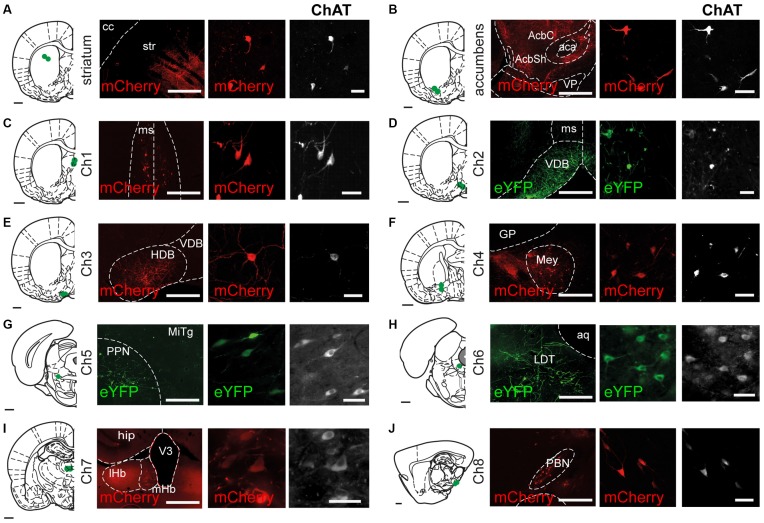
**Transduction of cholinergic neurons in different brain regions.** Coronal (Ch1 to Ch7) or sagittal (Ch8) sections showing the sites of cholinergic neuron transduction following injections of AAV2-DIO-EF1a-eYFP and AAV2-DIO-EF1a-mCherry into the striatum and the brain cholinergic nuclei (Ch1 to Ch8). Sections were immunolabeled for ChAT to outline cholinergic structures and verify the specificity of the reporter expression (eYFP, 92%; mCherry, 88%). eYFP or mCherry expression was observed in the dorsal striatum **(A)**, nucleus accumbens **(B)**, medial septum (Ch1; **C**), the vertical limb of the diagonal band of Broca (Ch2; **D**), the horizontal limb of the diagonal band of Broca (Ch3; **E**), the nucleus basalis of Meynert (Ch4; **F**), the pedunculopontine nucleus (Ch5; **G**), the laterodorsal tegmental nucleus (Ch6; **H**), the medial habenula (Ch7; **I**) and the parabigeminal nucleus (Ch8; **J**). All the injections were confined to their corresponding anatomical borders, as defined by Paxinos and Watson (2007). Abbreviations: aca, anterior commissure; aq, aqueduct; cc, corpus callosum; GP, globus pallidus; hip, hippocampus: HDB, horizontal limb of the diagonal band of the nucleus of Broca; LDT, laterodorsal tegmental nucleus; lHb, lateral habenula; Mey, nucleus basalis of Meynert; mHb, medial habenula; MITg, microcellular tegmental nucleus; ms, medial septum; ot, olfactory tubercle; PBN, parabigeminal nucleus; PPN, pedunculopontine nucleus; STR, striatum; V3, third ventricle; VDB, ventral limb of the diagonal band of the nucleus of Broca; VP, ventral pallidum. Scale bars: brain outlines, 1000 μm; low magnification panels (left), 500 μm; high magnification panels (center and right), 50 μm.

Transduction of cholinergic neurons in Ch1 to Ch8 areas in ChAT::Cre^+^ rats following the insertion of the reporter transgene produced strong and discrete eYFP or mCherry signals in neurons immunopositive for ChAT (Figure 1). Reporter labeling was observed in cell bodies, dendrites and local axons within the sites of injection. No differences were observed in the labeling produced by eYFP or mCherry (data not shown), nor in the labeling specificity among cholinergic structures. The labeling of cholinergic neurons with fluorescent reporters was confirmed by immunohistochemistry for ChAT in the following structures: dorsolateral striatum (Figure 1A), nucleus accumbens (Figure 1B), medial septum (Ch1; Figure 1C), the vertical limb of the diagonal band of the nucleus of Broca (Ch2; Figure 1D), the horizontal limb of the diagonal band of the nucleus of Broca (Ch3; Figure 1E), the nucleus basalis of Meynert (Ch4; Figure 1F), the pedunculopontine nucleus (Ch5; Figure 1G), the laterodorsal tegmental nucleus (Ch6; Figure 1H), the medial habenula (Ch7; Figure 1I) and the parabigeminal nucleus (Ch8; Figure 1J). Injections in the striatum and nucleus accumbens produced labeling of interneurons whose axons were contained within the striatal borders but extended ventrally or dorsally beyond the site of injection. However, no overlap between the axons from each region was detected, suggesting that the area of innervation of cholinergic axons is restricted within their functional domains. Medial septum injections were targeted to its mediodorsal region to avoid overlap with the diagonal band of the nucleus of Broca, which resulted in bilateral expression (Figure 1C). The vertical limb (Figure 1D) and the horizontal limb (Figure 1E) are very ventral structures surrounded by the ventral pallidum dorsally and the olfactory tubercle laterally; both show a high density of small cholinergic neurons. The nucleus of Meynert, situated ventral to the globus pallidus (GP), contained loosely distributed, small cholinergic neurons (Figure 1F), consistent with Mesulam et al. (1983a,b).

**Figure 2:**
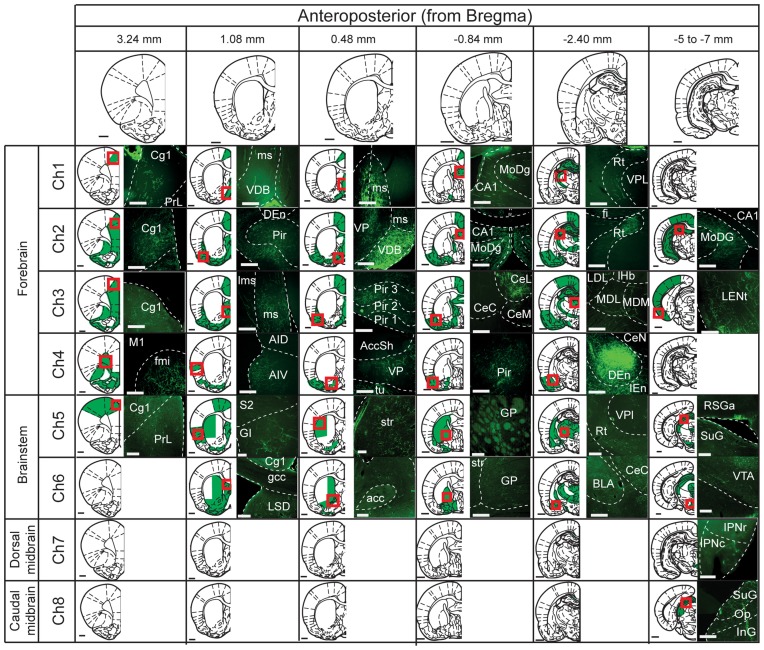
**Mapping of cholinergic axons.** Six representative coronal sections (anterior and posterior to bregma; columns) were selected to map the projections of the cholinergic neurons in the different cholinergic nuclei (Ch1 to Ch8; rows). For each cholinergic cell group, the rows depict schematic summaries where green shaded areas indicate high density of fluorescently-labeled axons. Red squares indicate the site where the fluorescent images on the right were obtained. Abbreviations (if not defined previously): Accsh, nucleus accumbens shell; AccC, nucleus accumbens core; AID, agranular insular dorsal cortex, AIV, agranular insular ventral cortex; BLA, basolateral amygdala; CA1, CA1 field of the hippocampus; CE, central amygdala (L, lateral; M, medial; C, capsular); Cg, cingulate cortex; Den, dorsal endopiriform nucleus; fmi, external capsule; GI, granular insular cortex; gcc, genu of the corpus callosum; GP, globus pallidus; IEn, intermediate endopiriform nucleus; InG, intermediate gray layer superior colliculus; IPN, interpeduncular nucleus (c, caudal; r, rostral); LDL, laterodorsal thalamic nucleus lateral part; LENt, lateral enthorinal cortex; Lms, lateral septum; LSD, lateral septum dorsal part; M, motor cortex; MD, mediodorsal thalamic nucleus (M, medial; L, lateral); MoDG, molecular layer dentate gyrus; Op, optic nerves layer superior colliculus; Pir, piriform cortex; PoDg, polymorph layer dentate gyrus; RSGa, retrospinal granular cortex; Rt, reticular thalamic nucleus; S, somatosensory cortex; SuG, superficial gray superior colliculus; VP, ventral pallidum; VPL, ventro-posterior thalamic nucleus lateral part; VTA, ventral tegmental area. Scale bars: brain outlines, 1000 μm; fluorescent images, 200 μm.

Medial septal (Ch1; Figure 2) cholinergic projections were mainly observed in the cingulate cortex (3+), the diagonal band of the nucleus of Broca (3+), the lateral septum (3+), the ventral pallidum (3+), hippocampus (4+) and the reticular thalamic nucleus (2+) (Nyakas et al., 1987; Kalén and Wiklund, 1989; Senut et al., 1989). No labeled axons were observed in the striatum or nucleus accumbens.

The axons and terminals of cholinergic neurons located in the vertical limb of the diagonal band of the nucleus of Broca (Ch2; Figure 2) were found predominantly in the medial prefrontal cortex (3+), the cingulate cortex (4+), the orbital cortex (4+), the motor cortex (4+), the piriform cortex (4+), the lateral and medial septum (4+), the ventral pallidum (3+), the amygdala (4+), the rostral and caudal hippocampus (4+), zona incerta (3+), mediodorsal and reticular thalamic nuclei (3+) (Kalén and Wiklund, 1989; Sarter and Bruno, 2000; Henny and Jones, 2008). Injections in Ch2 did not produce axon labeling in the striatum or the nucleus accumbens. However, a few projections were visible in the olfactory tubercle.

The cholinergic neurons of the horizontal limb of the diagonal band of the nucleus of Broca (Ch3; Figure 2) gave rise to similar projection patterns as Ch2 cholinergic neurons. eYFP-positive terminals were found mainly in the cingulate cortex (4+), medial prefrontal cortex (3+), motor cortex (3+), somatosensory cortex (3+), piriform cortex (4+), insular cortex (3+) and prelimbic cortex (3+). This population of neurons also gave rise to projections to the ventral pallidum (4+), the amygdala (4+), hippocampus (4+), and reticular and mediodorsal thalamic nuclei (3+) (Záborsky et al., 1986; Kalén and Wiklund, 1989; Gaykema et al., 1990; Gritti et al., 1997; Henny and Jones, 2008). Analysis of the Ch3-injected rats never revealed positive axons within the striatum or nucleus accumbens. However, *en-passage* fibers within the olfactory tubercle were observed.

The labeling of cholinergic neurons in the nucleus basalis of Meynert (Ch4; Figure 2) revealed cholinergic projections primarily to the orbital cortex (3+), the peduncular cortex (3+), the insular cortex (2+), the piriform cortex (3+), the ventral pallidum (4+) and the amygdala (4+) (Nagai et al., 1982; Woolf and Butcher, 1982; Pearson et al., 1983; Saper, 1984; Baskerville et al., 1993; Schauz and Koch, 1999; Záborszky et al., 2015). No labeled axons were observed in the striatum, the nucleus accumbens or the olfactory tubercle.

Injections in the PPN (Ch5; Figure 2) revealed weak axonal labeling in the anterior cingulate (1+), motor cortex (1+) and the insular cortex (2+). Much stronger labeling was observed in the ventral pallidum (3+), the medial and lateral septum (2+), the globus pallidus (2+), the amygdala (2+), the ventral and dorsal lateral thalamus (3+), the reticular thalamic nucleus (3+), the superior colliculus (3+), the dopaminergic ventral midbrain nuclei (3+), the LDT (3+) and the gigantocellular tegmental field (3+) (Mitani et al., 1988; Semba and Fibiger, 1992; Lavoie and Parent, 1994; Futami et al., 1995; Oakman et al., 1999; Mena-Segovia et al., 2004, 2008). Abundant labeled axons were observed in the dorsolateral striatum (3+), nucleus accumbens (3+) and olfactory tubercle (2+) (Dautan et al., 2014).

LDT-injected animals (Ch6; Figure 2) revealed labeled axons and terminals in the ventral pallidum (3+), medial and lateral septum (3+), globus pallidus (3+), amygdala (3+), reticular and medial thalamic nucleus (3+), inferior colliculus (2+), dorsal raphe (2+), gigantocellular tegmental field (3+) and the midbrain dopaminergic nuclei (3+) (Hallanger and Wainer, 1988; Motts et al., 2008; Holmstrand and Sesack, 2011). YFP-positive axons were observed in the dorsomedial striatum (2+), nucleus accumbens (3+) and olfactory tubercle (4+) (Dautan et al., 2014).

Medial habenula-injected animals (Ch7; Figure 2) showed a strong and discrete descending pathway that followed the fasciculus retroflexus and terminated in the interpeduncular nucleus (3+) (Cuello et al., 1978; Ren et al., 2011; Kobayashi et al., 2013). Animals injected in the parabigeminal nucleus (Ch8; Figure 2) showed an ascending pathway that spread densely in the inferior (3+) and superior colliculi (4+) (Mufson et al., 1986; Fitzpatrick et al., 1988). No rostrally projecting pathway was observed in animals injected either in the medial habenula or the parabigeminal nucleus; detailed examination of the striatum, accumbens and olfactory tubercle revealed no labeling within their borders.

The descriptions above comprise the main areas of innervation for each of the cholinergic groups.

### Retrograde Tracing

**Figure 3:**
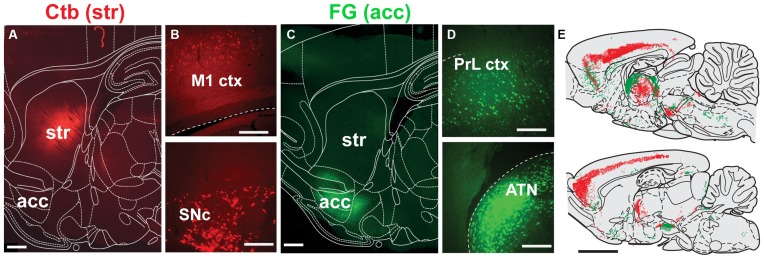
**Distribution of neurons projecting to the dorsal striatum and nucleus accumbens. (A,C)** Deposits of Ctb or FG were delivered into the dorsolateral striatum (STR) or nucleus accumbens (acc; this was alternated across animals), as shown in these examples from sagittal sections. **(B)** Representative examples of retrograde labeling in the motor cortex (M1 ctx) and substantia nigra pars compacta (SNc) following an injection in the dorsolateral striatum. **(D)** Representative examples of retrograde labeling in the prelimbic cortex (PrL ctx) and anterior thalamic nucleus (ATN) following an injection in the nucleus accumbens. **(E)** Mapping of retrogradely labeled neurons across the brain at two representative sagittal levels (lateral to bregma: 0.4 and 1.55 mm; each dot represent a positive cell body; FG, green; Ctb, red). Scale bars: **(A,C)** 1000 μm; **(B,D)** 200 μm; **(E)** 5000 μm.

Injections into the striatum and nucleus accumbens (Figures 3A,C) revealed widespread cell body labeling (Figure 3E), but predominantly in the deep layers of the motor, somatosensory and limbic cortices (Figures 3B,D). Dorsal striatum injections also produced a strong signal in the thalamus, predominantly in the anterior thalamic nucleus (ATN), the central-median, the parafascicular and the ventro-posterior nuclei. In contrast, nucleus accumbens injections led to retrograde labeling mainly in the parafascicular and medial thalamic nuclei. Further labeling following dorsal striatum injections was observed in the lateral substantia nigra compacta, whereas injections delivered in the nucleus accumbens produced labeling in the ventral tegmental area and medial substantia nigra pars compacta. Other nuclei that contained retrogradely labeled neurons include the GP, the ventral pallidum, dorsal raphe, ventral hypothalamus and locus coeruleus.

**Figure 4:**
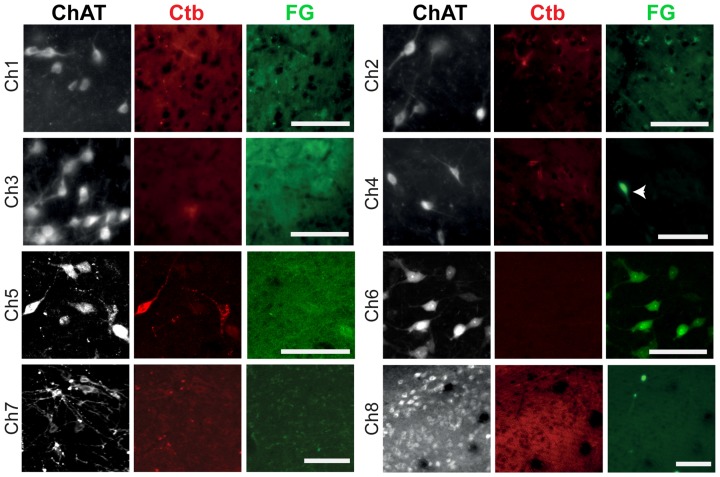
**Retrograde labeling in cholinergic cell groups Ch1–Ch8.** Following injections of the retograde tracers in the dorsolateral striatum, retrogradely-labeled neurons were absent in Ch1, Ch2, Ch3, Ch7 and Ch8, and present in Ch4, Ch5 and Ch6, but co-expression with ChAT immunolabeling was only observed in Ch5 and Ch6. In these examples, neurons projecting to the striatum were labeled by the Ctb (red) and neurons projecting to the nucleus accumbens were labeled by the FG (green). The arrow indicates a neuron containing FG that is ChAT-negative. Images from Ch1 to Ch7 were obtained from coronal sections, whereas the image from Ch8 was obtained from a sagittal section. Scale bars: Ch1 to Ch8, 100 μm.

Labeling of neurons within the borders of cholinergic structures (Ch1–Ch8) was observed in the nucleus of Meynert, PPN and LDT, with no neurons detected within the borders of Ch1, Ch2, Ch3, Ch7 or Ch8 groups (Figure 4). However, ChAT immunolabeling revealed that the majority of the retrogradely labeled neurons in the PPN and LDT are cholinergic (as reported in Dautan et al., 2014), whereas none of the retrogradely labeled neurons in the nucleus of Meynert was immunopositive for ChAT. These results further confirm the presence of a cholinergic projection from the PPN/LDT to the striatum and nucleus accumbens and shows that no other cholinergic nuclei contribute to the innervation to the striatal complex.

## Discussion

Following the recent report of an extensive, extrinsic source of acetylcholine in the striatum (Dautan et al., 2014) using novel tracing technologies, we evaluated here the possibility that the enhanced sensitivity of these techniques may reveal that other cholinergic structures across the brain contribute to the cholinergic transmission in the striatum. We targeted all eight cholinergic neuron groups to induce conditional anterograde labeling of cholinergic axons and observed that, besides the PPN and LDT (Ch5 and Ch6, respectively), no other cholinergic group gave rise to axons in the dorsal striatum or nucleus accumbens. A comprehensive mapping of the axon distribution of these structures shows presence of labeled axons in all of their known targets, thus corroborating the accuracy and specificity of our cholinergic transductions. Furthermore, analysis of retrogradely labeled neurons from the striatum and nucleus accumbens shows labeling of cholinergic neurons exclusively in the PPN and LDT. Our results thus confirm that no other sources of acetylcholine for the striatum exist besides the local interneurons and the cholinergic brainstem. These findings have important implications for understanding the role of the brainstem in striatal modulation and enable us to define more clearly and in clear functional terms the role of cholinergic systems in the modulation of striatal/basal ganglia circuits.

### Technical Considerations

The use of a Cre recombinase rat line together with AAV injections allowed us to target anatomically-restricted cholinergic groups and map their projections. However, virus injections necessary to reach a representative proportion of cholinergic neurons in each structure can potentially diffuse several hundred microns (Dautan et al., 2014), which becomes problematic for those cholinergic cell groups that form a continuum (e.g., Ch1, Ch2, Ch3). To overcome this difficulty, preventing the spread of the transduction over contiguous cholinergic groups and restricting the labeling to the defined borders of each structure, the volume of the injections was adjusted for each structure based on our preliminary assessments. Because our data is not used to evaluate the quantitative density of axons but it is rather based on the qualitative expression, the variations on the virus injection volumes are unlikely to affect the conclusions of this study.

The expression of eYFP can give rise to a low signal to noise ratio in thin axon shafts and small terminals, some of which can be photobleached rapidly and thus become difficult to detect during on-line analysis. In order to circumvent the possibility of false-negatives due to factors of this nature, we enhanced the YFP signal by immunostaining and performed the analysis off-line, thus minimizing the exposure of the tissue to the fluorescent light. It is worth noting that YFP labeling produces a complete labeling of axon terminals, as demonstrated previously by electron microscopy (Dautan et al., 2014), which may provide some advantage of sensitivity over conventional anterograde tracers and may account for the detection of previously unidentified targets. Additional validation of the data from the ChAT::Cre^+^ rats was obtained by the use of conventional retrograde tracers in wild-type rats. Our results showed that no retrogradely-labeled cholinergic cell bodies were observed in any of the cholinergic cell groups whose axons were absent from the striatal complex (i.e., Ch1–Ch4, Ch7 and Ch8), and in contrast, retrogradely-labeled cholinergic cell bodies were detected in the Ch5 (PPN) and Ch6 (LDT) regions, whose axons widely innervated the striatum and nucleus accumbens.

### Cholinergic Transmission in the Striatum

The effects of acetylcholine in the striatum are varied and complex (Sugita et al., 1991; Koós and Tepper, 2002; Goldberg et al., 2012). Muscarinic and nicotinic receptors are present at both the pre- and postsynaptic sites, and thus are able to regulate the activity of cortical, thalamic and dopaminergic terminals, as well as the activity of striatal projection neurons and different interneurons (Calabresi et al., 1998, 2000; Volpicelli-Daley et al., 2003; Bonsi et al., 2011; see review by Lim et al., 2014). The existence of an additional source of acetylcholine, provided by the brainstem, may be correlated to the diversity of acetylcholine receptors. Dissecting these two cholinergic systems thus becomes critical to fully understand the implications of cholinergic signaling in the striatum. Furthermore, because the cholinergic brainstem provides collaterals to the thalamus and the dopaminergic midbrain, two of the most important afferent systems to the striatum, it is likely that their influence on striatal circuits will be highly correlated with the thalamic and midbrain inputs. Such connectivity thus puts the PPN/LDT as an important station for striatal computations.

The lack of evidence from this advanced anatomical experimental approach of additional sources of acetylcholine to the striatum emphasizes the key role of the cholinergic brainstem for modulating striatal activity and basal ganglia function. Furthermore, because of the involvement of the PPN/LDT in neuropsychiatric disorders that affect predominantly the basal ganglia, and whose pathophysiology is associated with abnormal cholinergic transmission, such as Parkinson’s disease (Hirsch et al., 1987; Hall et al., 2014), Huntington’s disease (Picconi et al., 2006; Smith et al., 2006), progressive supranuclear palsy (Warren et al., 2005), and dystonia (Sciamanna et al., 2012), the evidence of a direct projection to the striatum opens new avenues for the interpretation of these abnormal processes and the challenges they pose. Further work is necessary to define the roles of this pathway in the activity of the basal ganglia in heath and disease.

## Author Contributions

DD and JMS conceived the project; DD performed the experiments; DD and HHB analyzed the data; DD, JPB, TVG and JMS wrote the article.

## Funding

This work was supported by the Medical Research Council UK (MC-UU-12020/1 to JPB). DD was funded by a University of Leicester PhD studentship. Access to data will be available on request.

## Conflict of Interest Statement

The authors declare that the research was conducted in the absence of any commercial or financial relationships that could be construed as a potential conflict of interest.

## References

[B2] Baskerville K. A. Chang H. T. Herron P. (1993). Topography of cholinergic afferents from the nucleus basalis of Meynert to representational areas of sensorimotor cortices in the rat. J. Comp. Neurol. 335, 552–562. 10.1002/cne.903350407 8227535

[B3] Bennett B. D. Callaway J. C. Wilson C. J. (2000). Intrinsic membrane properties underlying spontaneous firing in neostriatal cholinergic interneurons. J. Neurosci. 20, 8493–8503. 11069957 10.1523/JNEUROSCI.20-22-08493.2000 PMC6773196

[B4] Bolam J. P. Booth P. A. C. Hanley J. J. Bevan M. D. (2000). Synaptic organisation of the basal ganglia. J. Anat. 96, 527–542. 10.1046/j.1469-7580.2000.19640527.x 10923985 PMC1468095

[B5] Bonsi P. Cuomo D. Martella G. Madeo G. Schirinzi T. Puglisi F.. (2011). Centrality of striatal cholinergic transmission in basal ganglia function. Front. Neuroanat. 5:6. 10.3389/fnana.2011.00006 21344017 PMC3036975

[B6] Calabresi P. Centonze D. Gubellini P. Pisani A. Bernardi G. (1998). Endogeneous Ach enhances striatal NMDA-responses via M1-like muscarinic receptors and PKC activation. Eur. J. Neurosci. 10, 2887–2895. 10.1111/j.1460-9568.1998.00294.x 9758158

[B7] Calabresi P. Centoze D. Gubellini P. Marfia G. A. Pisani A. Sancesario G.. (2000). Synaptic transmission in the striatum: from plasticity to neurodegeneration. Prog. Neurobiol. 61, 231–265. 10.1016/s0301-0082(99)00030-1 10727775

[B9] Cuello C. A. Emson P. C. Paxinos G. Jessell T. (1978). Substance P containing and cholinergic projections from the habenula. Brain Res. 149, 413–429. 10.1016/0006-8993(78)90484-5 352479

[B10] Dautan D. Huerta-Ocampo I. Witten I. B. Deisseroth K. Bolam J. P. Gerdjikov T.. (2014). A major external source of cholinergic innervation of the striatum and nucleus accumbens originates in the brainstem. J. Neurosci. 34, 4509–4518. 10.1523/JNEUROSCI.5071-13.2014 24671996 PMC3965779

[B12] Ding J. B. Guzman J. N. Peterson J. D. Goldberg J. A. Surmeier D. J. (2010). Thalamic gating of corticostriatal signaling by cholinergic interneurons. Neuron 67, 294–307. 10.1016/j.neuron.2010.06.017 20670836 PMC4085694

[B13] Fitzpatrick D. Conley M. Luppino G. Matelli M. Diamond I. T. (1988). Cholinergic projections from the midbrain reticular formation and the parabigeminal nucleus to the lateral geniculate nucleus in the tree shrew. J. Comp. Neurol. 272, 43–67. 10.1002/cne.902720105 2454977

[B14] Futami T. Takakusaki K. Kitai S. T. (1995). Glutamatergic and cholinergic inputs from the pedunculopontine tegmental nucleus to dopamine neurons in the substantia nigra pars compacta. Neurosci. Res. 21, 331–342. 10.1016/0168-0102(94)00869-h 7777224

[B15] Gaykema R. P. Luiten P. G. Nyakas C. Traber J. (1990). Cortical projection patterns of the medial septum-diagonal band complex. J. Comp. Neurol. 293, 103–124. 10.1002/cne.902930109 2312788

[B16] Goldberg A. Ding J. B. Surmeier D. J. (2012). Muscarinic modulation of striatal function and circuitry. Handb. Exp. Pharmacol. 208, 223–241. 10.1007/978-3-642-23274-9_10 22222701

[B18] Gritti I. Mainville L. Mancia M. Jones B. E. (1997). GABAergic and other noncholinergic basal forebrain neurons, together with cholinergic neurons, project to the mesocortex and isocortex in the rat. J. Comp. Neurol. 383, 163–177. 10.1002/(sici)1096-9861(19970630)383:2<163::aid-cne4>3.3.co;2-t 9182846

[B19] Hall H. Reyes S. Landeck N. Bye C. Leanza G. Double K.. (2014). Hippocampal lewy pathology and cholinergic dysfunction are associated with dementia in Parkinson’s disease. Brain 137, 2493–2508. 10.1093/brain/awu193 25062696

[B20] Hallanger A. E. Wainer B. H. (1988). Ascending projections from the pedunculopontine tegmental nucleus and the adjacent mesopontine tegmentum in the rat. J. Comp. Neurol. 274, 483–515. 10.1002/cne.902740403 2464621

[B21] Hebb C. O. Silver A. (1961). Gradient of choline acetylase activity in nerve fibers. Nature 189, 123–125. 10.1038/189123a0 13712629

[B22] Henny P. Jones B. E. (2008). Projections from basal forebrain to prefrontal cortex comprise cholinergic, GABAergic and glutamatergic inputs to pyramidal cells or interneurons. Eur. J. Neurosci. 27, 654–670. 10.1111/j.1460-9568.2008.06029.x 18279318 PMC2426826

[B23] Hirsch E. C. Graybiel A. M. Duyckaerts C. Javoy-Agid F. (1987). Neuronal loss in the pedunculopontine tegmental nucleus in Parkinson disease and in progressive supranuclear palsy. Proc. Natl. Acad. Sci. U S A 84, 5976–5980. 10.1073/pnas.84.16.5976 3475716 PMC298986

[B24] Holmstrand E. C. Sesack S. R. (2011). Projections from the rat pedunculopontine and laterodorsal tegmental nuclei to the anterior thalamus and ventral tegmental area arise from largely separate populations of neurons. Brain Struct. Funct. 216, 331–345. 10.1007/s00429-011-0320-2 21556793 PMC3255475

[B26] Kalén P. Wiklund L. (1989). Projections from the medial septum and diagonal band of Broca to the dorsal and central superior raphe nuclei: a non-cholinergic pathway. Exp. Brain Res. 75, 401–416. 10.1007/bf00247947 2721618

[B27] Kobayashi Y. Sano Y. Vannoni E. Goto H. Suzuki H. Oba A.. (2013). Genetic dissection of medial habenula-interpeduncular nucleus pathway function in mice. Front. Behav. Neurosci. 7:17. 10.3389/fnbeh.2013.00017 23487260 PMC3594921

[B28] Koós T. Tepper J. M. (2002). Dual cholinergic control of fast-spiking interneurons in the neostriatum. J. Neurosci. 22, 529–535. 11784799 10.1523/JNEUROSCI.22-02-00529.2002 PMC6758683

[B29] Lavoie B. Parent A. (1994). Pedunculopontine nucleus in the squirrel monkey: projections to the basal ganglia as revealed by anterograde tract-tracing methods. J. Comp. Neurol. 344, 210–231. 10.1002/cne.903440204 8077458

[B30] Lim S. A. Kang U. J. McGehee D. S. (2014). Striatal cholinergic interneuron regulation and circuit effect. Front. Synaptic Neurosci. 6:22. 10.3389/fnsyn.2014.00022 25374536 PMC4204445

[B31] Macintosh F. C. (1941). The distribution of acetylcholine in the peripheral and the central nervous system. J. Physiol. Lond. 99, 436–442. 10.1113/jphysiol.1941.sp003913 16995263 PMC1394098

[B32] Mena-Segovia J. Bolam J. P. Magill P. J. (2004). Pedunculopontine nucleus and basal ganglia: distant relatives or part of the same family? Trends Neurosci. 27, 585–588. 10.1016/j.tins.2004.07.009 15374668

[B33] Mena-Segovia J. Sims H. M. Magill P. J. Bolam J. P. (2008). Cholinergic brainstem neurons modulate cortical gamma activity during slow oscillations. J. Physiol. Lond. 586, 2947–2960. 10.1113/jphysiol.2008.153874 18440991 PMC2517196

[B37] Mesulam M. M. (1990). Human brain cholinergic pathways. Prog. Brain Res. 84, 231–241. 10.1016/s0079-6123(08)60908-5 2267300

[B34] Mesulam M. M. Geula C. (1988). Nucleus basalis (Ch4) and cortical innervation in the human brain: observations based on distribution of acetylcholinesterase and choline acetyltransferase. J. Comp. Neurol. 275, 216–240. 10.1002/cne.902750205 3220975

[B35] Mesulam M. M. Mufson E. J. Levey A. J. Wainer B. H. (1983a). Cholinergic innervation of cortex by the basal forebrain: cytochemistry and cortical connections of the septal area, diagonal band nuclei, nucleus basalis (substantia innominata) and hypothalamus in the rhesus monkey. J. Comp. Neurol. 214, 170–197. 10.1002/cne.902140206 6841683

[B36] Mesulam M. M. Mufson E. J. Wainer B. H. Levey A. I. (1983b). Central cholinergic pathways in the rat: an overview based on an alternative nomenclature (Ch1–Ch6). Neuroscience 10, 1185–1201. 10.1016/0306-4522(83)90108-2 6320048

[B38] Mink J. W. (1996). The basal ganglia: focused selection and inhibition of competing motor programs. Prog. Neurobiol. 50, 381–425. 10.1016/s0301-0082(96)00042-1 9004351

[B39] Mitani A. Ito K. Hallanger A. E. Wainer B. H. Kataoka K. McCarley R. W. (1988). Cholinergic projections from the laterodorsal and pedunculopontine tegmental nuclei to the pontine gigantocellular tegmental field in the cat. Brain Res. 451, 397–402. 10.1016/0006-8993(88)90792-5 3251602

[B40] Motts S. D. Slusarczyk A. S. Sowick C. S. Schofield B. R. (2008). Distribution of cholinergic cells in guinea pig brainstem. Neuroscience 154, 186–195. 10.1016/j.neuroscience.2007.12.017 18222049 PMC2475650

[B41] Mufson E. J. Martin T. L. Mash D. C. Wainer B. H. Mesulam M. M. (1986). Cholinergic projections from the parabigeminal nucleus (Ch8) to the superior colliculus in the mouse: a combined analysis of horseradish peroxidase transport and choline acetyltransferase immunohistochemistry. Brain Res. 370, 144–148. 10.1016/0006-8993(86)91114-5 3708316

[B42] Nagai T. Kimura H. Maeda T. McGeer P. L. Peng F. McGeer E. G. (1982). Cholinergic projections from the basal forebrain of rat to the amygdala. J. Neurosci. 2, 513–520. 7069469 10.1523/JNEUROSCI.02-04-00513.1982 PMC6564247

[B43] Nakano K. Hasegawa Y. Tokushige A. Nakagawa S. Kayahara T. Mizuno N. (1990). Topographical projections from the thalamus, subthalamic nucleus and pedunculopontine tegmental nucleus to the striatum in the Japanese monkey, *Macaca fuscata*. Brain Res. 537, 54–68. 10.1016/0006-8993(90)90339-d 1707734

[B44] Nyakas C. Luiten P. G. M. Spencer D. G. Traber J. (1987). Detailed projection patterns of septal and diagonal band efferents to the hippocampus in the rat with emphasis on innervation of CA1 and Dentate gyrus. Brain Res. Bull. 18, 533–545. 10.1016/0361-9230(87)90117-1 3607523

[B45] Oakman S. A. Faris P. L. Cozzari C. Hartman B. K. (1999). Characterization of the extent of pontomesencephalic cholinergic neurons’ projections to the thalamus: comparison with projections to midbrain dopaminergic groups. Neuroscience 94, 529–547. 10.1016/s0306-4522(99)00307-3 10579214

[B47] Paxinos G. Watson C. (2007). The Rat Brain in Stereotaxic Coordinates. 6th Edn. San Diego: Elsevier Academic Press

[B48] Pearson R. C. A. Gatter K. C. Brodal P. Powell T. P. S. (1983). The projection of the basal nucleus of Meynert upon the neocortex in the monkey. Brain Res. 259, 132–136. 10.1016/0006-8993(83)91075-2 6824926

[B49] Picconi B. Passino E. Sgobio C. Bonsi P. Barone I. Ghiglieri V.. (2006). Plastic and behavioral abnormalities in experimental Huntington’s disease: a crucial role for cholinergic interneurons. Neurobiol. Dis. 22, 143–152. 10.1016/j.nbd.2005.10.009 16326108

[B50] Ren J. Qin C. Hu F. Tan J. Qiu L. Zhao S.. (2011). Habenula “cholinergic” neurons corelease glutamate and acetylcholine and activate postsynaptic neurons via distinct transmission modes. Neuron 69, 445–452. 10.1016/j.neuron.2010.12.038 21315256

[B52] Saper C. B. (1984). Organization of cerebral cortical afferent systems in the rat. J. Comp. Neurol. 222, 313–342. 10.1002/cne.902220302 6699210

[B51] Saper C. B. Loewy A. D. (1982). Projections of the pedunculopontine tegmental nucleus in the rat: evidence for additional extrapyramidal circuitry. Brain Res. 252, 367–372. 10.1016/0006-8993(82)90404-8 7150958

[B53] Sarter M. Bruno J. P. (2000). Cortical cholinergic inputs mediating arousal, attentional processing and dreaming: differential afferent regulation of the basal forebrain by telencephalic and brainstem afferents. Neuroscience 95, 933–952. 10.1016/s0306-4522(99)00487-x 10682701

[B54] Schauz C. Koch M. (1999). Lesions of the nucleus basalis magnocellularis do not impair prepulse inhibition and latent inhibition of fear-potentiated startle in the rat. Brain Res. 815, 98–105. 10.1016/s0006-8993(98)01134-2 9974127

[B55] Sciamanna G. Tassone A. Mandolesi G. Puglisi F. Ponterio G. Martella G.. (2012). Cholinergic dysfunction alters synaptic integration between thalamostriatal and corticostriatal inputs in DYT1 dystonia. J. Neurosci. 32, 11991–12004. 10.1523/JNEUROSCI.0041-12.2012 22933784 PMC3471539

[B56] Semba K. Fibiger H. C. (1992). Afferent connections of the laterodorsal and the pedunculopontine tegmental nuclei in the rat: a retro- and antero-grade transport and immunohistochemical study. J. Comp. Neurol. 323, 387–410. 10.1002/cne.903230307 1281170

[B57] Senut M. C. Menetry D. Lamour Y. (1989). Cholinergic and peptidergic projections from the medial septum and the nucleus of the diagonal band of Broca to dorsal hippocampus, cingulate cortex and olfactory bulb: a combined wheat-germ agglutinin horseradish peroxidase gold immunohistochemical study. Neuroscience 30, 385–404. 10.1016/0306-4522(89)90260-1 2473418

[B58] Smith R. Chung H. Rundquist S. Maat-Schieman M. L. C. Colgan L. Englund E.. (2006). Cholinergic neuronal defect without cell loss in Huntington’s disease. Hum. Mol. Genet. 15, 3119–3131. 10.1093/hmg/ddl252 16987871

[B59] Smith Y. Parent A. (1986). Differential connections of caudate nucleus and putamen in squirrel monkey (*Saimiri sciureus*). Neuroscience 18, 347–371. 10.1016/0306-4522(86)90159-4 3736862

[B60] Sugita S. Uchimura N. Jiang Z. G. North R. A. (1991). Distinct muscarinic receptors inhibit release of γ-aminobutyric acid and excitatory amino acids in mammalian brain. Proc. Natl. Acad. Sci. U S A 88, 2608–2611. 10.1073/pnas.88.6.2608 1672454 PMC51282

[B61] Volpicelli-Daley L. A. Hrabovska A. Duysen E. G. Ferguson S. M. Brakely R. D. Lockridge A.. (2003). Altered striatal function and muscarinic cholinergic receptors in acetylcholinesterase knockout mice. Mol. Pharmacol. 64, 1309–1316. 10.1124/mol.64.6.1309 14645660

[B62] Wall N. R. De La Parra M. Callaway E. M. Kreitzer A. C. (2013). Differential innervation of direct- and indirect-pathway striatal projection neurons. Neuron 79, 347–360. 10.1016/j.neuron.2013.05.014 23810541 PMC3729794

[B63] Wang Z. Kai L. Day M. Ronesi J. Yin H. H. Ding J.. (2006). Dopaminergic control of corticostriatal longterm synaptic depression in medium spiny neurons is mediated by cholinergic interneurons. Neuron 50, 443–452. 10.1016/j.neuron.2006.04.010 16675398

[B64] Warren N. M. Piggott M. A. Perry E. K. Burn D. J. (2005). Cholinergic in progressive supranuclear palsy. Brain 128, 239–249. 10.1093/brain/awh391 15649952

[B65] Witten I. B. Steinberg E. E. Lee S. Y. Davidson T. J. Zalocusky K. A. Brodsky M.. (2011). Recombinase-driver rat lines: tools, techniques and optogenetic application to dopamine-mediated reinforcement. Neuron 72, 721–733. 10.1016/j.neuron.2011.10.028 22153370 PMC3282061

[B66] Woolf N. J. Butcher L. L. (1982). Cholinergic projections to the basolateral amygdala: a combined Evan’s blue and acetylcholinesterase analysis. Brain Res. Bull. 8, 751–763. 10.1016/0361-9230(82)90102-2 6182963

[B67] Woolf N. J. Eckenstein F. Butcher L. L. (1984). Cholinergic systems in the rat brain. Brain Res. Bull. 13, 751–784. 6532518 10.1016/0361-9230(84)90236-3

[B68] Záborsky L. Carlsen J. Brashear H. R. Heimer L. (1986). Cholinergic and GABAergic afferents to the olfactory bulb with special emphasis on the projections in the nucleus of the horizontal limb of the diagonal band. J. Comp. Neurol. 243, 488–509. 10.1002/cne.902430405 3512629

[B69] Záborszky L. Csordas A. Mosca K. Kim J. Gielow M. R. Vadasz C.. (2015). Neurons in the basal forebrain project to the cortex in a complex topographic organization that reflects corticocortical connectivity patterns: an experimental study based on retrograde tracing and 3D reconstruction. Cereb. Cortex 25, 118–137. 10.1093/cercor/bht210 23964066 PMC4259277

